# ACTH-dependent Cushing Syndrome with No Peripheral Response But a Marked Petrosal Sinus ACTH Response to Desmopressin

**DOI:** 10.1210/jcemcr/luae092

**Published:** 2024-05-27

**Authors:** Martin Guay-Gagnon, Ran Cheng, Catherine Beauregard, André Lacroix

**Affiliations:** Division of Endocrinology, Department of Medicine and Research Center, Centre hospitalier de l’Université de Montréal, Montréal, QC H2X 0A9, Canada; Division of Endocrinology, Department of Medicine and Research Center, Centre hospitalier de l’Université de Montréal, Montréal, QC H2X 0A9, Canada; Division of Endocrinology, Department of Medicine and Research Center, Centre hospitalier de l’Université de Montréal, Montréal, QC H2X 0A9, Canada; Division of Endocrinology, Department of Medicine and Research Center, Centre hospitalier de l’Université de Montréal, Montréal, QC H2X 0A9, Canada

**Keywords:** Cushing syndrome, desmopressin, ACTH, inferior petrosal sinus sampling, prolactin, cabergoline

## Abstract

Desmopressin is increasingly used for the diagnosis of Cushing disease (CD) since corticotropin-releasing hormone became unavailable. We report the case a 32-year-old man who presented with overt Cushing syndrome. Morning blood cortisol, ACTH, 1 mg dexamethasone suppression test, 24-hour urinary free cortisol, and bedtime salivary cortisol were highly variable, reaching markedly elevated values. Intravenous desmopressin administration produced no ACTH or cortisol increase. Pituitary magnetic resonance imaging, thoracic computed tomography, and DOTATATE positron emission tomography scan identified no lesion. Inferior petrosal sinus sampling (IPSS) with desmopressin stimulation resulted in elevated central-to-peripheral ACTH ratio and prolactin co-secretion, while peripheral ACTH remained stable. No corticotroph tumor was identified on pituitary surgery pathology. Hypercortisolism persisted postoperatively. Cabergoline was initiated, after which the patient rapidly developed transient severe adrenal insufficiency (AI). Bilateral adrenalectomy was performed in view of persistent hypercortisolism. This is an unusual case of petrosal sinus ACTH response to desmopressin without any peripheral response, suggesting a central source of ACTH. Thus, desmopressin should still be used during IPSS in patients with no peripheral response. It is unclear whether the AI episode resulted from a combination of nadir of cyclic hypercortisolism, partial apoplexy, and response to cabergoline of an occult corticotroph tumor.

## Introduction

Identifying the source of ACTH-dependent Cushing syndrome (CS) can be a diagnostic challenge. Peripheral ACTH stimulation by corticotropin-releasing hormone (CRH), as well as central ACTH stimulation by CRH during inferior petrosal sinus sampling (IPSS) are valuable tools to distinguish between a pituitary and ectopic source of ACTH. Now that CRH is no longer available, desmopressin is increasingly used and has been found to be a suitable alternative (1). The mechanism by which desmopressin stimulates ACTH secretion in CD may result from overexpression of AVPR1B receptors as well as ectopic AVPR2 receptors in corticotroph adenomas, which is more prevalent in tumors harboring *USP8* mutations (2, 3). Here we describe a case of ACTH-dependent CS with no peripheral ACTH response but a marked inferior petrosal sinus ACTH response to desmopressin administration. Following lack of remission after pituitary surgery, therapy with cabergoline was initiated. The patient developed acute adrenal insufficiency (AI), which was rapidly reversible following cabergoline discontinuation. Bilateral adrenalectomy was performed to treat his persistent highly variable hypercortisolism.

## Case Presentation

A 32-year-old man was referred to our center for evaluation and therapy of CS. Over the previous 2 years he had developed diabetes, hypertension, hypokalemia, osteoporosis, obesity, and overt Cushingoid appearance. Biochemical testing showed highly variable and marked hypercortisolism: morning cortisol varied between 369 and 8065 nmol/L (13 and 292 µg/dL) (normal: 185–624 nmol/L; 6.7–22.6 µg/dL), bedtime salivary cortisol between 208 and 371 nmol/L (7.5 and 13.4 µg/dL) (normal: < 5 nmol/L; < 0.18 µg/dL), and 24-hour urinary free cortisol between 32 499 and 50 663 nmol/day (11 775 and 18 356 µg/day) (normal:<120 nmol/day; <43.5 µg/day), and a 1 mg dexamethasone suppression test result was 462 nmol/L (16.7 µg/dL) (normal:<50 nmol/L; <1.8 µg/dL). ACTH was also variable, ranging between 3.3 and 63 pmol/L (15 and 286 pg/mL) (normal: 2–11 pmol/L; 9-50 pg/mL) **(**Table 1**).**

**Table 1.** Initial biochemical testing for Cushing syndrome

Test	Results	Normal values
Morning cortisol	369–8065 nmol/L (13–292 µg/dL)	185–624 nmol/L (6.7–22.6 µg/dL)
Bedtime salivary cortisol	208–371 nmol/L (7.5–13.4 µg/dL)	<5 nmol/L (<0.18 µg/dL)
24-hour UFC	32 499–50 663 nmol/day (11 775–18 356 µg/day)	<120 nmol/day (<43.5 µg/day)
1 mg DST cortisol value	462 nmol/L (16.7 µg/dL)	<50 nmol/L (<1.8 µg/dL)
ACTH	3.3–63 pmol/L (15–286 pg/mL)	2–11 pmol/L (9–50 pg/mL)

Abbreviations: DST, dexamethasone suppression test; UFC, urinary free cortisol.

## Diagnostic Assessment

**Figure 1.**
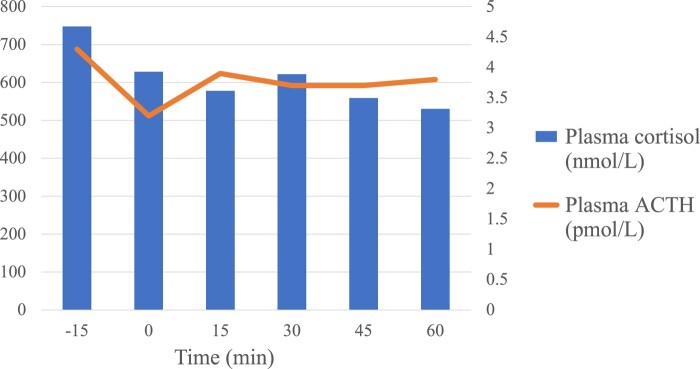
Desmopressin (10 µg intravenous at 0 time point) stimulation test results.

A 4 mg intravenous (IV) dexamethasone suppression test showed partial cortisol suppression: cortisol was 527 nmol/L (19 µg/dL) at baseline, reached a nadir of 255 nmol/L (9 µg/dL) 4 hours after dexamethasone infusion, and rebounded early to 1850 nmol/L (67 µg/dL) after 7 hours and to 3750 nmol/L (136 μg/dL) the following morning. ACTH was 3.5 pmol/L (15.9 pg/mL) at baseline, 18.2 pmol/L (82.6 pg/mL) after 7 hours, and 7.8 pmol/L (35.4 pg/mL) the following morning. A desmopressin (10 μg IV) stimulation test showed no response: ACTH remained stable from 3.8 pmol/L (17.3 pg/mL) to 3.9 pmol/L and cortisol from 688 nmol/L (25 μg/dL) to 622 nmol/L (23 μg/dL) **(**Fig. 1). Pituitary magnetic resonance imaging, thoracic computed tomography, and DOTATATE positron emission tomography scan identified no lesion, and abdominal computed tomography showed bilateral adrenal hyperplasia. IPSS performed with desmopressin suggested a pituitary origin with central-to-peripheral (C/P) ACTH ratios that rose from 7.1 to 34.2 on the left. Right C/P ACTH ratios were equivocal (basal: 1.19 and stimulated: 3.19), possibly due to poor selectivity in the right petrosal sinus with a C/P prolactin ratio of 1.33. Peripheral ACTH remained low and stable, between 2.7 and 3.7 pmol/L (12.3 and 16.8 pg/mL). Cortisol was low at the time of the IPSS (105-146 nmol/L; 3.8–5.3 µg/dL). Prolactin increased by 83% in the left IPSS after desmopressin, while peripheral prolactin remained stable **(**Table 2**).**

**Table 2.** **Inferior petrosal sinus sampling with desmopressin 10** **μg intravenous injection (0 time point) showing high central-to-peripheral ACTH gradient on the left**

Time	Right petrosal sinus ACTH	Left petrosal sinus ACTH	Peripheral plasma ACTH	Right petrosal sinus prolactin	Left petrosal sinus prolactin	Peripheral plasma prolactin	Plasma cortisol
Basal ref. range	**—**	—	2–11 pmol/L (9–50 pg/mL)	**—**	**—**	2.5–17.4 µg/L (2.5–17.4 ng/mL)	<276 nmol/L (<10 µg/dL)*^a^*
−5 minutes	3.5 pmol/L (15.9 pg/mL)	19.3 pmol/L (87.6 pg/mL)	3.0 pmol/L (13.6 pg/mL)	20.6 µg/L (20.6 ng/mL)	91.9 µg/L (91.9 ng/mL)	15.0 µg/L (15.0 ng/mL)	105 nmol/L (3.8 µg/dL)
0 minutes	3.3 pmol/L (15.0 pg/mL)	21.0 pmol/L (95.4 pg/mL)	2.7 pmol/L (12.3 pg/mL)	17.9 µg/L (17.9 ng/mL)	85.6 µg/L (85.6 ng/mL)	14.0 µg/L (14.0 ng/mL)	118 nmol/L (4.3 µg/dL)
3 minutes	10.2 pmol/L (46.3 pg/mL)	109.5 pmol/L (497.2 pg/mL)	3.2 pmol/L (14.5 pg/mL)	23.9 µg/L (23.9 ng/mL)	162.5 µg/L (162.5 ng/mL)	13.4 µg/L (13.4 ng/mL)	106 nmol/L (3.8 µg/dL)
5 minutes	6.1 pmol/L (27.7 pg/mL)	43.9 pmol/L (199.3 pg/mL)	3.7 pmol/L (16.8 pg/mL)	24.5 µg/L (24.5 ng/mL)	108.6 µg/L (108.6 ng/mL)	14.5 µg/L (14.5 ng/mL)	127 nmol/L (4.6 µg/dL)
10 minutes	3.6 pmol/L (16.3 pg/mL)	16.9 pmol/L (76.7 pg/mL)	3.2 pmol/L (14.5 pg/mL)	19.1 µg/L (19.1 ng/mL)	59.5 µg/L (59.5 ng/mL)	14.9 µg/L (14.9 ng/mL)	110 nmol/L (4.0 µg/dL)
30 minutes			3.1 pmol/L (14.1 pg/mL)			14.1 µg/L (14.1 ng/mL)	146 nmol/L (5.3 µg/dL)
45 minutes			2.7 pmol/L (12.3 pg/mL)			13.5 µg/L (13.5 ng/mL)	141 nmol/L (5.1 µg/dL)
60 minutes			3.0 pmol/L (13.6 pg/mL)			14.4 µg/L (14.4 ng/mL)	129 nmol/L (4.7 µg/dL)

^*a*^Reference range at the time of sampling (2:05 Pm).

## Treatment

A pituitary exploration was performed, during which the surgeon resected a suspected tumor on the left. However, pathology showed normal pituitary tissue with rare Crooke's cells, suggesting cortisol excess. Hypercortisolism persisted postoperatively with partial diabetes insipidus. Oral desmopressin and cabergoline 0.5 mg twice weekly were initiated.

## Outcome and Follow-up

Two weeks later, the patient was admitted to the emergency unit with nausea, vomiting, and lethargy. Upon evaluation, the patient was found to be hypotensive (80/50 mmHg), hyponatremic (132 mmol/L, normal: 135–145 mmol/L), and mildly hypoglycemic (3.7 mmol/L [67 mg/dL], normal range: 4.0–11.1 nmol/L; 72–200 mg/dL). A septic workup showed no evidence of infection, and morning cortisol was 233 nmol/L (8.4 μg/dL), abnormally low in this context. AI was diagnosed, and the patient's condition promptly resolved with IV hydrocortisone and fluid resuscitation. Cabergoline was stopped, and highly variable hypercortisolism recurred a few days later. With the anticipated difficulty of treating medically a highly variable ACTH-secreting tumor and the patient's preference for an efficacious and permanent therapy, bilateral adrenalectomy was performed, which resulted in rapid clinical remission. In the immediate postoperative period, the patient developed diplopia and vertical nystagmus, and a right cerebellar stroke was confirmed on cerebral imaging with expected postoperative changes in the sella turcica. An embolic source was suspected, but further investigations were negative. The patient did not have long-term deficits. Adrenal pathology showed bilateral hyperplasia consistent with chronic ACTH excess. On hydrocortisone replacement (25 mg/day), his most recent ACTH was 21.2 pmol/L (96.3 pg/mL).

## Discussion

This is an unusual case of highly positive petrosal sinus ACTH stimulation by desmopressin without any peripheral response, suggesting a central source of ACTH in a patient with ACTH-dependant CS. Three similar cases with a lack of peripheral ACTH response in the initial desmopressin test but with a central response during IPSS were reported; however, simultaneous peripheral ACTH results were not provided (4). According to a recent meta-analysis, the desmopressin stimulation test has a sensitivity of 88% for discriminating CD from normal patients with clinical or biochemical features suggestive of CS, indicating nonetheless a false-negative rate of 12% (5). The usefulness of peripheral desmopressin test in differentiating CD from ectopic ACTH secretion (EAS) is less clear, with varying results across different studies, as some ectopic tumors also respond to desmopressin. The combination of desmopressin with CRH may yield better results (1, 6). A meta-analysis comparing desmopressin and CRH stimulation during IPSS found that desmopressin had high sensitivity (96%) and specificity (100%), similar to CRH, for distinguishing CD from EAS (7).

In our case, the discrepancy between the absence of peripheral ACTH response during the desmopressin stimulation test and the marked central ACTH response during IPSS cannot be solely attributed to variations due to cyclic ACTH-dependent CS, because peripheral ACTH during IPSS also showed no response to desmopressin. The low peripheral ACTH during IPSS suggests that it was during a nadir of cyclic CS. We hypothesize that the short and transient increase in ACTH secretion could be detected in the petrosal sinus but was too small to significantly influence systemic levels. Thus, the use of desmopressin during IPSS should still be attempted in patients with no prior peripheral response.

Unfortunately, pituitary exploration did not identify a corticotroph adenoma confirmed by pathology. This raises the possibility that this patient had an occult ectopic source of ACTH with cyclic secretion. The IPSS clearly suggested a pituitary source of ACTH, but was done during a nadir of disease as demonstrated by the low basal peripheral cortisol and ACTH. Under CRH stimulation, IPSS results could be explained by a temporary loss of negative feedback of cortisol on pituitary ACTH secretion in the case of a cyclic EAS caught during a nadir. However, desmopressin is less likely to stimulate normal corticotroph cells (5, 6). No literature was found on IPSS results with desmopressin on healthy subjects. However, patients with pseudo-Cushing and healthy subjects show minimal to no peripheral ACTH response to desmopressin, with some variability across studies (5, 6). Prolactin increased by 88% in the left petrosal sinus after desmopressin. Prolactin is usually measured during IPSS to assess the selectivity of catheterization. A subset of patients with CD cosecrete prolactin in response to CRH during IPSS. Proposed mechanisms include paracrine effects of proopiomelanocortin-derived peptides such as beta-endorphins on lactotroph cells and direct secretion of prolactin by corticotroph cells (8). We consider that the prolactin cosecretion observed in this patient during IPSS supports the presence of a small unidentified corticotroph adenoma.

A few possible causes may underlie the acute AI episode following cabergoline initiation. A nadir of cyclic CS could be the main contributing factor (9). Pituitary apoplexy is a rare complication of dopamine agonists, especially reported in the treatment of prolactinomas (10). Unfortunately, no cerebral imaging or complete pituitary hormone panel was done during the episode. However, the absence of visible pituitary adenoma on magnetic resonance imaging and the rapid recurrence of hypercortisolism upon cabergoline discontinuation are not in favor of apoplexy. An excellent response of nonvisible corticotropinoma to cabergoline causing AI is a plausible explanation; a subset of corticotroph adenomas express D2 receptors and their stimulation by dopamine agonists inhibits ACTH secretion (11). A retrospective study on the efficacy of cabergoline in CD including 53 patients found normalization of hypercortisolism in 40% of patients within 12 months, with 5 patients developing AI, some of them with doses as low as 0.5 mg/week (11).

In conclusion, we report the case of a patient with cyclic ACTH-dependent CS who showed no peripheral ACTH response to desmopressin stimulation but a marked increase of central ACTH and prolactin following desmopressin during IPSS. This case demonstrates that desmopressin stimulation during IPSS is useful even in the absence of a peripheral ACTH response. It also illustrates the complexity of investigating and treating cyclic CS. IPSS was done during a nadir of cortisol secretion that renders its interpretation difficult. No tumor was found on pituitary pathology, and the patient developed an episode of acute AI under therapy with cabergoline; bilateral adrenalectomy resolved the hypercortisolism but will require long-term follow-up with adequate steroid replacement and surveillance for progression of presumed occult pituitary corticotroph tumor or, alternatively, occult ectopic ACTH-secreting neuroendocrine tumor.

## Learning Points

- Desmopressin is a suitable alternative to CRH for ACTH stimulation in the investigation of CS.
- Desmopressin should be used for ACTH stimulation during IPSS even in patients with no prior peripheral ACTH response to desmopressin.
- For IPSS to be reliable in cyclic CS, it should be undertaken during a period of active hypercortisolism but may be difficult when daily erratic secretion is present.
- Bilateral adrenalectomy remains an efficacious and permanent treatment in cases of refractory hypercortisolism of uncertain etiology.
- Further research is necessary to better understand the mechanisms underlying prolactin secretion in petrosal sinus following ACTH secretagogues and if this could be a predictor of dopamine agonist response in CD.

## Data Availability

Original data generated and analyzed during this study are included in this published article.
